# Bis{2-[(*E*)-benzyl­imino­meth­yl]-4,6-dibromo­phenolato-κ^2^ *N*,*O*}cobalt(II)

**DOI:** 10.1107/S1600536808032303

**Published:** 2008-10-15

**Authors:** Wei Jiang, Gui-Di Mo, Lie Jin

**Affiliations:** a School of Chemistry and Life Sciences, Maoming University, Guandu Second Road 139, Maoming 525000, Guangdong, People’s Republic of China

## Abstract

In the title compound, [Co(C_14_H_10_Br_2_NO)_2_], the Co^II^ ion is coordinated by an O and an N atom from two equivalent 2-[(*E*)-benzyl­imino­meth­yl]-4,6-dibromo­phenolate ligands, displaying a distorted tetra­hedral geometry. The Co^II^ ion occupies a special position on a twofold rotation axis and thus the mol­ecular symmetry of the complex is *C*_2_. The two phenolate rings are perpendicular [89.8 (3)°].

## Related literature

For general background on the applications of Schiff bases, see: Vigato *et al.* (2007 ▶).
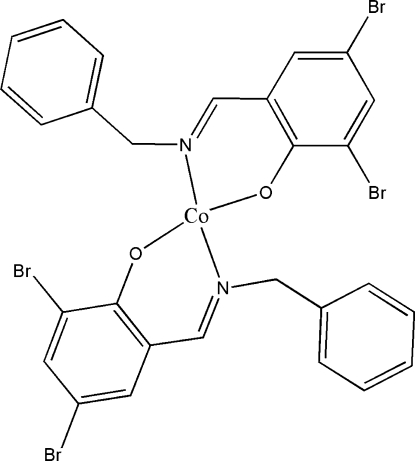


## Experimental

### 

#### Crystal data

[Co(C_14_H_10_Br_2_NO)_2_]
*M* *_r_* = 795.03
Monoclinic, 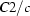

*a* = 23.875 (3) Å
*b* = 4.8190 (6) Å
*c* = 24.209 (3) Å
β = 105.8730 (1)°
*V* = 2679.1 (6) Å^3^
*Z* = 4
Mo *K*α radiation
μ = 6.64 mm^−1^
*T* = 296 (2) K
0.30 × 0.26 × 0.22 mm

#### Data collection

Bruker SMART APEXII CCD diffractometer
Absorption correction: multi-scan (*SADABS*; Sheldrick, 2000 ▶) *T*_min_ = 0.165, *T*_max_ = 0.232
11046 measured reflections
3094 independent reflections
2627 reflections with *I* > 2σ(*I*)
*R*_int_ = 0.027

#### Refinement

*R*[*F* ^2^ > 2σ(*F* ^2^)] = 0.052
*wR*(*F* ^2^) = 0.172
*S* = 1.07
3094 reflections
168 parameters
H-atom parameters constrained
Δρ_max_ = 0.98 e Å^−3^
Δρ_min_ = −1.46 e Å^−3^

### 

Data collection: *APEX2* (Bruker, 2004 ▶); cell refinement: *SAINT* (Bruker, 2004 ▶); data reduction: *SAINT*; program(s) used to solve structure: *SHELXS97* (Sheldrick, 2008 ▶); program(s) used to refine structure: *SHELXL97* (Sheldrick, 2008 ▶); molecular graphics: *XP* in *SHELXTL* (Sheldrick, 2008 ▶); software used to prepare material for publication: *XP* in *SHELXTL*.

## Supplementary Material

Crystal structure: contains datablocks global, I. DOI: 10.1107/S1600536808032303/kp2194sup1.cif

Structure factors: contains datablocks I. DOI: 10.1107/S1600536808032303/kp2194Isup2.hkl

Additional supplementary materials:  crystallographic information; 3D view; checkCIF report

## Figures and Tables

**Table:** 

Co1—O1	1.935 (4)
Co1—N1	2.005 (4)

**Table:** 

O1^i^—Co1—O1	126.8 (2)
O1^i^—Co1—N1	94.16 (17)
O1—Co1—N1	113.29 (17)
N1—Co1—N1^i^	117.1 (3)

Symmetry code: (i) 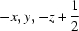.

**Fig. 1.**
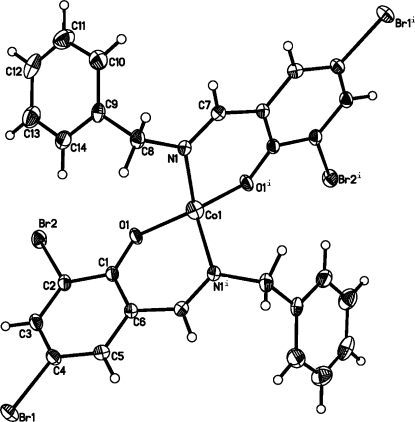
The structure of (I), showing displacement ellipsoids drawn at the 30% probability level. [Symmetry code: (i) -x, y, -z+0.5]

## supplementary crystallographic information

### Comment

The Schiff bases are widely employed as ligands in coordination chemistry. The advantages of Schiff bases enable their use in the synthesis of metal complexes of interest in bioinorganic chemistry, catalysis, encapsulation, transport and separation processes, and magnetochemistry (Choi & Jeon, 2003). Salicylaldehyde and its derivatives are useful carbonyl precursors for the synthesis of a large variety of Schiff bases. In this paper we report on a new cobalt(II) complex (I).

In the title complex Co^II^ atom is tetrahedrally coordinated by two O atoms and two N atoms from two 2-((E)-(benzylimino)methyl)-4,6-dibromophenol bidentate chelating ligand. The Co1—O1 distance of 1.935 (4) Å is shorter than the distance of Co1—N1 (2.005 (4) Å) (Table 1). The dihedral angle between two phenol rings is 89.8 (3)°.

### Experimental

To a solution containing 2 mmol (0.738 g) 2-((E)-(benzylimino)methyl)-4,6-dibromophenol dissolved in 20 mL ethanol, 1 mmol of CoCl_2_.6H_2_O (0.238 g) were added, and the resulting mixture was stirred for about 1 h. The slow evaporisation of the solvent after about 3 d yielded dark brown single crystals. Yield: 51.4%. Calcd. for C_28_H_20_Br_4_CoN_2_O_2_:  C, 42.30; H, 2.54; N, 3.52; Found: C, 42.24; H, 3.41; N,3.46%.

### Refinement

All H atoms were located from difference Fourier syntheses, H atoms from the C—H groups were placed in geometrically idealized positions and constrained to ride on their parent atoms (C—H = 0.93 Å, 0.96 Å, 0.97 Å) and *U*_iso_(H) values equal to 1.2 *U*_eq_(C).

### Crystal data

**Table:**

[Co(C_14_H_10_Br_2_NO)_2_]	*F*(000) = 1540
*M**_r_* = 795.03	*D*_x_ = 1.971 Mg m^−^^3^
Monoclinic, *C*2/*c*	Mo *K*α radiation, λ = 0.71073 Å
Hall symbol: -C 2yc	Cell parameters from 2269 reflections
*a* = 23.875 (3) Å	θ = 1.0–27.7°
*b* = 4.8190 (6) Å	µ = 6.64 mm^−^^1^
*c* = 24.209 (3) Å	*T* = 296 K
β = 105.873 (1)°	Block, brown
*V* = 2679.1 (6) Å^3^	0.30 × 0.26 × 0.22 mm
*Z* = 4	

### Data collection

**Table:**

Bruker SMART APEXII CCD diffractometer	3094 independent reflections
Radiation source: fine-focus sealed tube	2627 reflections with *I* > 2σ(*I*)
graphite	*R*_int_ = 0.027
φ and ω scans	θ_max_ = 27.7°, θ_min_ = 1.8°
Absorption correction: multi-scan (SADABS; Sheldrick, 2000)	*h* = −30→30
*T*_min_ = 0.165, *T*_max_ = 0.232	*k* = −6→6
11046 measured reflections	*l* = −30→31

### Refinement

**Table:**

Refinement on *F*^2^	Primary atom site location: structure-invariant direct methods
Least-squares matrix: full	Secondary atom site location: difference Fourier map
*R*[*F*^2^ > 2σ(*F*^2^)] = 0.052	Hydrogen site location: inferred from neighbouring sites
*wR*(*F*^2^) = 0.172	H-atom parameters constrained
*S* = 1.07	*w* = 1/[σ^2^(*F*_o_^2^) + (0.1045*P*)^2^ + 20.5054*P*] where *P* = (*F*_o_^2^ + 2*F*_c_^2^)/3
3094 reflections	(Δ/σ)_max_ < 0.001
168 parameters	Δρ_max_ = 0.98 e Å^−^^3^
0 restraints	Δρ_min_ = −1.45 e Å^−^^3^

### Special details

**Table:**

Geometry. All esds (except the esd in the dihedral angle between two l.s. planes) are estimated using the full covariance matrix. The cell esds are taken into account individually in the estimation of esds in distances, angles and torsion angles; correlations between esds in cell parameters are only used when they are defined by crystal symmetry. An approximate (isotropic) treatment of cell esds is used for estimating esds involving l.s. planes.
Refinement. Refinement of F^2^ against ALL reflections. The weighted R-factor wR and goodness of fit S are based on F^2^, conventional R-factors R are based on F, with F set to zero for negative F^2^. The threshold expression of F^2^ > 2sigma(F^2^) is used only for calculating R-factors(gt) etc. and is not relevant to the choice of reflections for refinement. R-factors based on F^2^ are statistically about twice as large as those based on F, and R- factors based on ALL data will be even larger.

### Fractional atomic coordinates and isotropic or equivalent isotropic displacement parameters (Å^2^)

**Table:**

	*x*	*y*	*z*	*U*_iso_*/*U*_eq_	
Co1	0.0000	0.9824 (2)	0.2500	0.0481 (3)	
Br1	0.25733 (3)	0.73207 (15)	0.14570 (3)	0.0446 (2)	
Br2	0.06423 (3)	1.45190 (13)	0.09822 (2)	0.0389 (2)	
O1	0.04168 (16)	1.1622 (8)	0.20173 (16)	0.0316 (8)	
N1	−0.06936 (19)	0.7651 (9)	0.20554 (18)	0.0269 (9)	
C1	0.0898 (2)	1.0738 (10)	0.1927 (2)	0.0260 (10)	
C2	0.1095 (2)	1.1793 (11)	0.1464 (2)	0.0285 (10)	
C3	0.1595 (2)	1.0865 (12)	0.1339 (2)	0.0313 (11)	
H3A	0.1707	1.1621	0.1032	0.038*	
C4	0.1925 (2)	0.8813 (13)	0.1672 (2)	0.0321 (11)	
C5	0.1772 (2)	0.7738 (12)	0.2136 (2)	0.0336 (11)	
H5A	0.2005	0.6395	0.2364	0.040*	
C6	0.1268 (2)	0.8649 (12)	0.2269 (2)	0.0281 (10)	
C7	−0.1144 (2)	0.7334 (12)	0.2242 (2)	0.0309 (11)	
H7A	−0.1425	0.6106	0.2037	0.037*	
C8	−0.0702 (3)	0.6166 (12)	0.1512 (2)	0.0327 (11)	
H8A	−0.0934	0.4492	0.1482	0.039*	
H8B	−0.0309	0.5631	0.1517	0.039*	
C9	−0.0954 (3)	0.7997 (11)	0.1000 (2)	0.0320 (11)	
C10	−0.1555 (3)	0.8139 (16)	0.0759 (3)	0.0483 (16)	
H10A	−0.1807	0.7055	0.0901	0.058*	
C11	−0.1773 (4)	0.9955 (19)	0.0296 (3)	0.059 (2)	
H11A	−0.2174	1.0061	0.0137	0.071*	
C12	−0.1433 (4)	1.1523 (16)	0.0075 (3)	0.0555 (19)	
H12A	−0.1593	1.2715	−0.0229	0.067*	
C13	−0.0827 (4)	1.1356 (17)	0.0309 (3)	0.0533 (17)	
H13A	−0.0581	1.2426	0.0157	0.064*	
C14	−0.0597 (3)	0.9608 (14)	0.0764 (3)	0.0420 (14)	
H14A	−0.0195	0.9504	0.0915	0.050*	

### Atomic displacement parameters (Å^2^)

**Table:**

	*U*^11^	*U*^22^	*U*^33^	*U*^12^	*U*^13^	*U*^23^
Co1	0.0443 (5)	0.0596 (6)	0.0428 (5)	0.000	0.0163 (4)	0.000
Br1	0.0289 (3)	0.0637 (4)	0.0467 (4)	0.0012 (2)	0.0197 (3)	−0.0043 (3)
Br2	0.0460 (4)	0.0389 (3)	0.0348 (3)	0.0015 (2)	0.0160 (3)	0.0099 (2)
O1	0.0320 (19)	0.039 (2)	0.0299 (19)	0.0049 (16)	0.0186 (16)	0.0067 (16)
N1	0.027 (2)	0.033 (2)	0.021 (2)	0.0010 (16)	0.0071 (17)	0.0003 (16)
C1	0.028 (2)	0.030 (2)	0.022 (2)	−0.0039 (19)	0.0092 (19)	−0.0012 (18)
C2	0.033 (3)	0.031 (3)	0.024 (2)	−0.006 (2)	0.011 (2)	−0.0022 (19)
C3	0.032 (3)	0.038 (3)	0.028 (3)	−0.010 (2)	0.014 (2)	−0.005 (2)
C4	0.026 (2)	0.042 (3)	0.032 (3)	−0.004 (2)	0.012 (2)	−0.006 (2)
C5	0.027 (3)	0.044 (3)	0.029 (3)	0.001 (2)	0.006 (2)	0.000 (2)
C6	0.026 (2)	0.038 (3)	0.022 (2)	−0.002 (2)	0.0078 (19)	−0.001 (2)
C7	0.027 (3)	0.042 (3)	0.023 (2)	−0.004 (2)	0.006 (2)	−0.003 (2)
C8	0.041 (3)	0.034 (3)	0.025 (2)	0.006 (2)	0.013 (2)	−0.002 (2)
C9	0.045 (3)	0.031 (3)	0.023 (2)	0.001 (2)	0.013 (2)	−0.0074 (19)
C10	0.043 (4)	0.061 (4)	0.042 (4)	0.007 (3)	0.014 (3)	0.004 (3)
C11	0.048 (4)	0.076 (5)	0.046 (4)	0.016 (4)	0.000 (3)	0.003 (4)
C12	0.084 (6)	0.051 (4)	0.025 (3)	0.005 (4)	0.005 (3)	0.001 (3)
C13	0.065 (5)	0.056 (4)	0.036 (3)	−0.011 (4)	0.010 (3)	0.006 (3)
C14	0.044 (3)	0.052 (4)	0.029 (3)	−0.007 (3)	0.008 (3)	0.000 (2)

### Geometric parameters (Å, °)

**Table:**

Co1—O1^i^	1.935 (4)	C6—C7^i^	1.441 (7)
Co1—O1	1.935 (4)	C7—C6^i^	1.441 (7)
Co1—N1	2.005 (4)	C7—H7A	0.9300
Co1—N1^i^	2.005 (4)	C8—C9	1.505 (8)
Br1—C4	1.902 (5)	C8—H8A	0.9700
Br2—C2	1.888 (6)	C8—H8B	0.9700
O1—C1	1.298 (6)	C9—C14	1.388 (8)
N1—C7	1.285 (7)	C9—C10	1.396 (9)
N1—C8	1.493 (7)	C10—C11	1.404 (11)
C1—C2	1.424 (7)	C10—H10A	0.9300
C1—C6	1.442 (7)	C11—C12	1.323 (12)
C2—C3	1.383 (7)	C11—H11A	0.9300
C3—C4	1.379 (8)	C12—C13	1.405 (11)
C3—H3A	0.9300	C12—H12A	0.9300
C4—C5	1.376 (8)	C13—C14	1.375 (10)
C5—C6	1.399 (8)	C13—H13A	0.9300
C5—H5A	0.9300	C14—H14A	0.9300
			
O1^i^—Co1—O1	126.8 (2)	N1—C7—C6^i^	127.9 (5)
O1^i^—Co1—N1	94.16 (17)	N1—C7—H7A	116.0
O1—Co1—N1	113.29 (17)	C6^i^—C7—H7A	116.0
O1^i^—Co1—N1^i^	113.29 (17)	N1—C8—C9	110.5 (4)
O1—Co1—N1^i^	94.16 (17)	N1—C8—H8A	109.6
N1—Co1—N1^i^	117.1 (3)	C9—C8—H8A	109.6
C1—O1—Co1	125.4 (3)	N1—C8—H8B	109.6
C7—N1—C8	116.2 (5)	C9—C8—H8B	109.6
C7—N1—Co1	121.4 (4)	H8A—C8—H8B	108.1
C8—N1—Co1	122.3 (4)	C14—C9—C10	118.5 (6)
O1—C1—C2	120.9 (5)	C14—C9—C8	121.1 (6)
O1—C1—C6	124.4 (4)	C10—C9—C8	120.5 (6)
C2—C1—C6	114.7 (4)	C9—C10—C11	118.6 (7)
C3—C2—C1	123.3 (5)	C9—C10—H10A	120.7
C3—C2—Br2	118.2 (4)	C11—C10—H10A	120.7
C1—C2—Br2	118.6 (4)	C12—C11—C10	123.0 (7)
C4—C3—C2	119.6 (5)	C12—C11—H11A	118.5
C4—C3—H3A	120.2	C10—C11—H11A	118.5
C2—C3—H3A	120.2	C11—C12—C13	118.8 (7)
C5—C4—C3	120.6 (5)	C11—C12—H12A	120.6
C5—C4—Br1	120.0 (5)	C13—C12—H12A	120.6
C3—C4—Br1	119.3 (4)	C14—C13—C12	120.0 (7)
C4—C5—C6	120.6 (5)	C14—C13—H13A	120.0
C4—C5—H5A	119.7	C12—C13—H13A	120.0
C6—C5—H5A	119.7	C13—C14—C9	121.2 (7)
C5—C6—C7^i^	115.6 (5)	C13—C14—H14A	119.4
C5—C6—C1	121.2 (5)	C9—C14—H14A	119.4
C7^i^—C6—C1	123.2 (5)		

Symmetry codes: (i) −*x*, *y*, −*z*+1/2.

## References

[bb1] Bruker (2004). *APEX2* and *SAINT* Bruker AXS Inc., Madison, Wisconsin, USA.

[bb3] Sheldrick, G. M. (2000). *SADABS* University of Göttingen, Germany.

[bb4] Sheldrick, G. M. (2008). *Acta Cryst.* A**64**, 112–122. 10.1107/S0108767307043930 18156677

[bb2] Vigato, P.A., Tamburini, S. & Bertolo, L. (2007). *Coord. Chem. Rev.* **251**, 1311–1316.

